# Retraction Note: Flexible Field Emitter for X-ray Generation by Implanting CNTs into Nickel Foil

**DOI:** 10.1186/s11671-019-3234-1

**Published:** 2020-01-07

**Authors:** Bin Sun, Yan Wang, Guifu Ding

**Affiliations:** National Key Laboratory of Micro/Nano Fabrication Technology, School of Electronic Information and Electrical Engineering, Shanghai Jiao Tong University, Shanghai, 200240 People’s Republic of China

**Retraction Note: Nanoscale Res Lett (2016) 11: 326**

**https://doi.org/10.1186/s11671-016-1523-5**

The Editors-in-Chief have retracted this article [1] because it significantly overlaps with a previously published article by the same authors [2].

The authors agree with this retraction.
